# Long-Term Results of the Mini Maze Standalone Bi-Atrial Surgical Ablation: A 10-Year Follow-Up

**DOI:** 10.3390/jcm13082195

**Published:** 2024-04-10

**Authors:** Greta Radauskaite, Gediminas Rackauskas, Svetlana Danilenko, Vilius Janusauskas, Audrius Aidietis

**Affiliations:** 1 Department of Cardiovascular Medicine, Faculty of Medicine, Vilnius University, 01513 Vilniaus, Lithuania; gediminas.rackauskas@santa.lt (G.R.); vilius.janusauskas@santa.lt (V.J.); audrius.aidietis@santa.lt (A.A.); 2 Vilnius University Hospital Santaros Klinikos, 08661 Vilniaus, Lithuania; 3 Department of Mathematical Statistics, Vilnius Gediminas Technical University, 10223 Vilniaus, Lithuania; svetlana.danilenko@vilniustech.lt

**Keywords:** atrial fibrillation, surgical ablation, maze procedure

## Abstract

**Background:** One way to treat atrial fibrillation is through surgical ablation. However, the literature only provides information on patient follow-up results for up to 5 years. **Methods:** In order to assess long-term monitoring data over ten years, this retrospective study included 58 patients with paroxysmal or persistent atrial fibrillation who underwent Mini Maze surgical ablation at Santaros Clinics between 1 February 2009 and 1 June 2014. The follow-up time after surgery was 144 ± 48 months. We evaluated the absence of atrial fibrillation, echocardiographic and clinical parameters, and EHRA score. **Results:** Sinus rhythm remained in 69.4%, 75.5%, 55.6%, and 44.1% of patients with paroxysmal AF, and 68,2%, 59.1%, 50%, and 41.9% of patients with persistent AF (*p* = 0.681). In the post-operative period, one patient (1.7%) had a transient ischemic attack, and another patient (1.7%) had a thoracotomy for post-operative bleeding. A total of 20% of patients were diagnosed with a post-operative respiratory tract infection. EHRA scores showed that patients’ quality of life improved after they underwent Mini Maze surgical ablation. **Conclusions:** Despite AF recurrences after surgery, quality of life remains better than before surgery, showing that Mini Maze surgery is an effective and safe second-line treatment method for atrial fibrillation.

## 1. Introduction

Atrial fibrillation is the most commonly diagnosed heart rhythm disorder [1]. Its prevalence has reached epidemic levels worldwide. In Europe, 11 million people have been diagnosed with AF, and 886,000 new patients are confirmed each year [2]. Atrial fibrillation (AF) is predicted to affect 6–12 million people in the USA by 2050 and 17.9 million people in Europe by 2060 [1,3]. AF has a profound negative effect on patients’ health, as it can lead to stroke, heart failure, depression, and a reduced quality of life [3]. Moreover, AF is associated with an increased risk of cardiac and total mortality [3]. Symptomatic AF reduces patients’ ability to work, increases disability, and increases the frequency of hospital visits. This strains the healthcare system and increases the cost of treatment. The first-line treatment for AF is often medical treatment [4]; however, in many cases, medical treatment does not have the desired clinical effect. Catheter ablation is an alternative to AF medical treatment [4], and surgical approaches have also been developed to treat AF, particularly for patients requiring concomitant cardiac surgery or those refractory to medical and catheter ablation treatments [4].

The improvements in surgical ablation techniques and technologies over the past decade have increased and have been shown to be relatively safe and effective, but many reports are limited to short follow-up periods [5,6].

The Cox-Maze procedure currently involves the isolation of pulmonary vein and linear atrium lesions and amputation of the left atrial appendage [7]. The procedure has become the gold standard for eliminating AF [7].

Many studies report the outcomes of Mini Maze post-operative follow-up for up to 5 years [8,9,10]. This study aimed to assess the long-term outcomes extending 10 years and beyond. We assessed the incidence of rhythm disturbances in the late post-operative period and the incidence of repeat procedures for AF. This study also aimed to investigate the impact of atrial fibrillation recurrence on patients’ quality of life.

## 2. Materials and Methods

### 2.1. Population

This retrospective study included 58 patients who underwent the Maze operation between 1 February 2009 and 1 June 2014. We only included patients who underwent standalone Maze surgery, excluding those who underwent other cardiac surgical procedures. All patients experienced symptoms of AF. There were two groups of patients: patients with paroxysmal AF and patients with persistent AF. Paroxysmal AF was intermittent, resolving spontaneously or within 7 days after medical therapy or electrical cardioversion. Persistent atrial fibrillation was defined as a rhythm disturbance lasting more than 7 days. Surgery was recommended due to ineffective medical treatment using class I or III antiarrhythmic drugs or previous unsuccessful AF ablation.

Post-procedural follow-up time was 144 ± 48 months after the Maze procedure. Patients underwent scheduled follow-up appointments at Santaros clinics at one and six months and one, two, four, and ten years after the procedure. A blanking period of 3 months after the procedure was implemented.

All patients agreed to participate in this study and signed an informed consent form. This study was approved by the Lithuanian Bioethics Committee (Approval Code: Nr.2020/9-1270-744; Approval Date: 10 September 2020).

### 2.2. Patient Follow-Up

**Figure 1:**
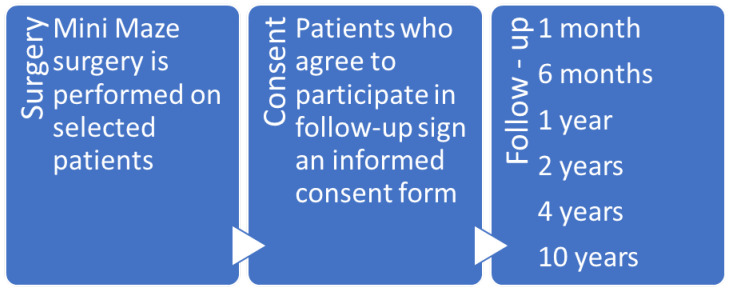
Study flow chart.

In this study, we collected information about patients’ medical conditions, 1 and 6 months and 1, 2, 4, and 10 years after discharge from the hospital (Figure 1).

Mini Maze surgery was performed on 127 patients, and 91 of them signed a consent form to participate in our study. Some patients could not be contacted for post-operative follow-up due to a change in their contact details, and some patients refused to come for follow-up visits due to the COVID-19 pandemic. Patients were enrolled in the study by the team that performed the procedure. Follow-up was carried out in the outpatient Cardiology Department of Santara Clínics.

We collected patient demographic characteristics such as age and sex and assessed all patients for stroke risk by determining a CHA2DS2-VASc score. We assessed the patient’s clinical symptoms of heart failure by NYHA functional class. During each visit, we asked about changes to medical records, and we conducted an electrocardiogram for each patient. A physical examination was also performed at each visit. We performed Holter monitoring 6 months and 1, 2, 4, and 10 years after the Mini Maze procedure. AF recurrence was determined using an electrocardiogram (ECG) or 24 h Holter monitoring, detecting episodes of atrial fibrillation or atrial flutter lasting more than 30 s [11]. The AF or atrial flutter episodes were terminated with medical antiarrhythmic treatment, electrical cardioversion, or a pulmonary vein isolation procedure/atrial flutter catheter ablation. Some patients were left with permanent AF; for some of them, a permanent pacemaker was implanted, and patients with tachysystole AF underwent AV node modification.

All patients received anticoagulation therapy with oral anticoagulants: warfarin or non-vitamin K antagonists (apixaban, rivaroxaban, edoxaban, or dabigatran) for 6 months. Anticoagulants were discontinued in patients with CHA2DS2-VASc scores less than 2, if on 24 h Holter monitoring no AF was detected, or if transoesophageal echocardioscopy showed left atrial appendage ligation.

We evaluated the European Heart Rhythm Association (EHRA) score. EHRA score is a proven and validated tool for assessing the symptoms of atrial fibrillation as follows [12]:

- EHRA I—‘No symptoms’.
- EHRA II—‘Mild symptoms’; normal daily activity.
- EHRA III—‘Severe symptoms’; limited daily activity.
- EHRA IV—‘Disabling symptoms’; unable to carry out daily activities.

An echocardiogram was performed during each patient’s visit. Left atrium (LA) sizes were measured using 2D ultrasound methods. We looked at every patient’s LA inferior–superior diameter, LA medial–lateral diameter, and LA volume index using a four-camera image. There was one lead cardiologist who performed cardiac ultrasound scans on the study patients. The rest of the research team consisted of three physicians who knew the protocol for the patients in this study and performed the tests in the absence of the principal investigator. However, all images were additionally evaluated by the leading cardiologist.

All Mini Maze procedures were carried out by the same team.

### 2.3. Surgical Technique

#### 2.3.1. Patient Positioning and Anaesthesia

The patient was positioned supine with their arms at their sides. We placed a foam roll approximately 5 cm in diameter under the patient’s shoulders to improve exposure for the surgical team. General anaesthesia with a double-diameter endotracheal tube was used during the procedure. Routine cardiac monitoring, including electrocardiography and invasive radial arterial and central venous pressure, was performed throughout the procedure. A Foley catheter was inserted to monitor diuresis. Saturation and pulmonary ventilation parameters were assessed throughout the operation. External defibrillation pads were placed in conventional positions. Transoesophageal electrocardiography was performed during the procedure to assess the status of the left atrial appendage and the presence of a thrombus.

#### 2.3.2. Right-Side Access

Left lung single-lung ventilation was performed. A 4 cm long incision was made in the fourth intercostal space on the right side of the chest between the midclavicular and midaxillary lines. The surgical team carefully dissected the chest layers and opened the parietal pleura. A pericardiotomy was performed approximately 1–2 cm anterior to the phrenic nerve. After the pericardiotomy, pericardial clips were placed to hold the heart closer to the incision. The oblique pericardial sinus was entered using blunt dissection between the inferior pulmonary vein and the inferior vein cava. A Navigator^®^ device (Medtronic, Inc., Dublin, Ireland) was inserted between the superior vena cava and the superior right pulmonary vein, through which a wire was passed. Tension on the pericardial retraction sutures was released. The wire was pushed into the transverse duck, and the Navigator^®^ was pulled out. The gastric probe was inserted into the diagonal duck.

#### 2.3.3. Left-Side Access

A 5 cm thoracotomy incision was made in the third intercostal space between the anterior and midaxillary lines. Right lung single-lung ventilation was performed. We dissected the chest layers until the parietal pleura was opened. The pericardiotomy was performed about 2 cm above the phrenic nerve. After the pericardiotomy, pericardial clips were placed to hold the heart closer to the incision.

The wire in the transverse antrum and the gastric probe in the diagonal antrum were withdrawn to the left side of the thorax. Using the plastic guides provided in the Medtronic Cardioblate^®^ Gemini^®^ Surgical Ablation System (Medtronic, Inc.) kit, the bipolar ablation forceps were advanced with one part above the pulmonary veins and the other below. Each ablation was completed when the machine confirmed that the transmural lesion had been reached. Three ablations were performed around the pulmonary veins on the left side of the chest. Three more ablations around the pulmonary veins were performed on the right side. The Medtronic Cardioblate^®^ Gemini^®^ was used to create ablation lines from the pulmonary vein isolation line to the right atrial appendage, on the right atrium’s surface towards the atrioventricular vein, and around the inferior vena cava. After every procedure, the exit block of the PVI box created by the ablation lines was checked. Each pulmonary vein above the ablation line was paced with a rate higher than the patient’s heart rate, and 20 mA stimuli were used to confirm the exit block. If PVI was not achieved, individual ablation of each of the four pulmonary veins was performed.

#### 2.3.4. Right Atrial Ablation

After we achieved PVI, we performed right atrial ablation. Additional ablation lines were created on the right atrium using the bipolar ablation device: a longitudinal line from the right atrium appendage targeting the intra-atrial septum between the right pulmonary veins, a line from the lateral part of the right atrium toward the tricuspid valve annulus, and a circular line at the ostium of the inferior vena cava. These ablation lines were directed around the cavotricuspid perimeter. Although there is ample evidence that the triggers originate from the superior vena cava, the superior vena cava was not ablated to avoid damage to the sinus node. Linear blockade after ablation was not checked.

**Scheme 1:**
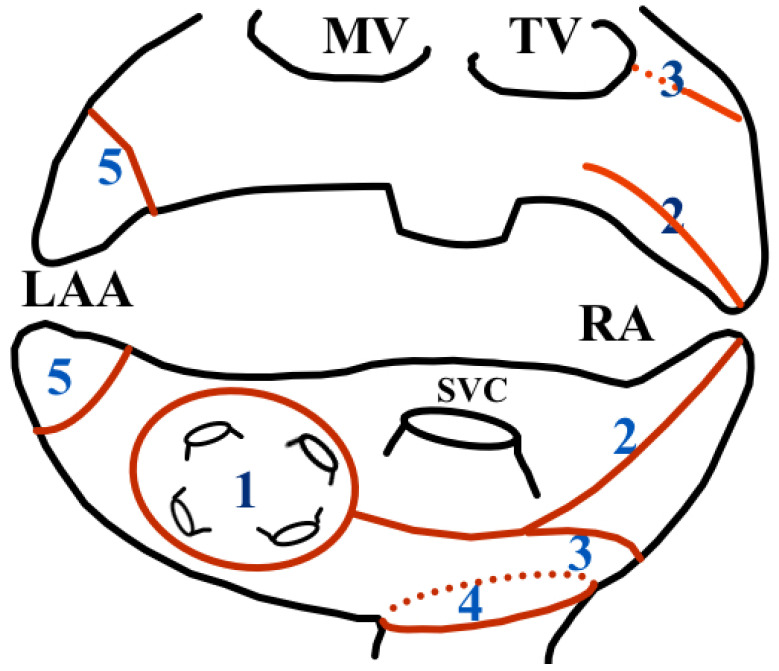
Ablation lines.

Ablation lines are shown in Scheme 1.

Ablation lines:

1. Pulmonary vein isolation (“Box lesion”).
2. From RA appendage to “box lesion”.
3. From mid of RA to “box lesion”.
4. IVC ostia.

Additional procedures:

5. Ligation of left atrial appendage and cutting of the Ligament of Marshall.

#### 2.3.5. Additional Procedures

The operation was accompanied by a left atrial appendage ligation and a cut in Marshall’s ligament. The left atrial appendage was reattached with a 2/0 polyester suture using a knot pusher. Two ligatures were placed, one at the base of the atrial appendage and the other approximately 7 mm distal to the first ligature. The end of the atrial appendage was cut to remove any remaining blood, and the ligature’s quality was checked.

### 2.4. Statistical Analysis

The collected data were processed with SPSS Statistics software (version 26.0. IMB Corp., Armonk, NY, USA). The Shapiro–Wilk test was used to normalise the quantitative variables. Student’s *t*-test for paired samples was used to compare quantitative variables between two independent groups. The Friedman Test, a non-parametric test, was used for analysing 3 or more dependent and 2 independent samples; the indicators were compared using the Wilcoxon Signed Rank Test. For the 2 independent samples, the indicators were compared using the Mann–Whitney Test. Cross-tabulations were calculated for the qualitative variables. Frequency tables were crosstabulated for the qualitative variables. Pearson’s Chi-Square or the non-parametric Mann–Whitney U test was used to test hypotheses for comparing categorical variables between groups.

A *p*-value less than 0.05 was considered significant.

## 3. Results

We performed a long-term follow-up of 58 patients who underwent Mini Maze surgery. The average follow-up time was 144 ± 48 months. The average length of stay in the ICU was 2 days, and the average length in the hospital was 14 ± 6 days.

The average patient’s age was 50.74 ± 8.93 years. The average duration of history of atrial fibrillation was 72.53 ± 42 months. Most patients who underwent surgery were male, accounting for 84.5% (49) of the study group. Only 15.5% (9) of patients were women.

The average patient‘s CHA2DS2-VASc score was 1.28 ± 1.04.

A total of 36 (62.1%) patients had paroxysmal AF, while 22 (37.9%) had persistent AF.

The main preoperative characteristics of patients are given in Table 1. Patient characteristics are given according to their AF type: paroxysmal or persistent.

Patients with persistent AF had a higher LA volume index than patients with paroxysmal AF. There were no other significant differences between the two groups of patients.

We also evaluated LA characteristics in the same paroxysmal and persistent AF patient groups before surgical treatment and 4 and 10 years after surgery. These data are presented in Table 2.

The results show a significant difference between paroxysmal and persistent atrial fibrillation groups in left atrial volume index measurements. The LA volume index is higher in the persistent AF group both before and 10 years after surgery (38.89 ± 10.66 vs. 45.57 ± 14.50; *p* = 0.05 and 51.02 ± 12.04 vs. 59.58 ± 13.45, respectively; *p* = 0.016).

We also evaluated LA values before, 4 years, and 10 years after the procedure in terms of whether or not the rhythm disturbances recurred in the post-operative period. These data are reflected in Table 3.

The results show that patients who were free of AF in the post-operative period for 10 years had a significantly lower LA volume index (51.06 ± 12.22 vs. 58.21 ± 13.44, respectively; *p* = 0.035) and LA inferior–superior (67.0 ± 6.1 vs. 71.2 ± 6.1, respectively; *p* = 0.020) and medial–lateral (52.9 ± 5.1 vs. 59.9 ± 4.9, respectively; *p* = 0.027) diameters. However, these parameters did not differ significantly in the pre-procedural period in these two groups. Patients with a bigger left atrial volume index had increased recurrences of atrial fibrillation after surgery.

**Figure 2:**
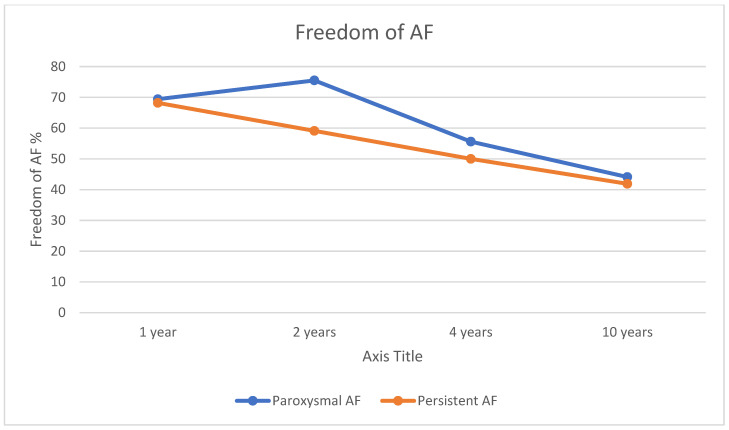
Freedom of AF after surgery.

One year after the Mini Maze surgery, no AF relapses were found in 69% of patients; 2 years after the procedure, 69% had sinus rhythm. A total of 53.4% of patients had no relapse of AF 4 years after surgery, and 43% had sinus rhythm 10 years after surgery. No significant difference was observed between the incidence of rhythm disturbances in the paroxysmal and persistent AF groups. Sinus rhythm remained in 69.4%, 75.5%, 55.6%, and 44.1% of patients with paroxysmal AF, and 68.2%, 59.1%, 50%, and 41.9% of patients with persistent AF (*p* = 0.681) (Figure 2). The freedom of arrhythmia was similar in both paroxysmal and persistent AF patient groups.

During the follow-up period, 15.5% (9) of patients had repeated pulmonary vein isolation procedures, and 24% (14) had atrial flutter ablation procedures. A total of 25% (14) of patients had persistent atrial fibrillation after a 10-year follow-up; 5% (1) of them underwent permanent pacemaker implantation and atrioventricular node modification due to uncontrolled tachysystole despite taking medication. A total of 6% (three) of patients had permanent pacemaker implantation due to sinus node dysfunction.

The following complications were also observed in the post-operative period: One patient (1.7%) had a transient ischaemic attack. Neurological symptoms were unexpressed and regressed within 24 h. As a result, the patient’s hospital stay was not prolonged. One patient (1.7%) had a thoracotomy for post-operative bleeding. A total of 20% of patients were diagnosed with a post-operative respiratory tract infection, confirmed via radiographs, fever, and elevated inflammatory parameters. Intravenous antibiotics were administered to treat the respiratory tract infection, and the patients were discharged for outpatient treatment when their inflammatory parameters returned to normal.

**Figure 3:**
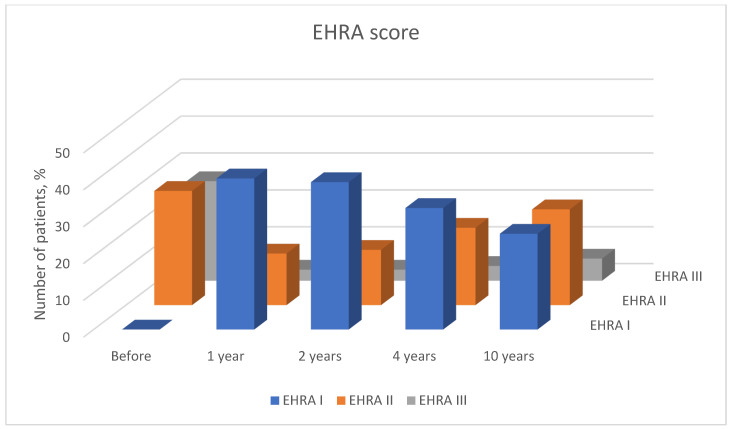
EHRA score dynamics during the follow-up period.

One of the treatment goals was to improve patients’ quality of life. We also evaluated the quality of life by assessing the EHRA classes before Mini Maze surgery and at each follow-up visit. Before the surgery, 31 patients (54%) had an EHRA II score and 27 (46%) had an EHRA III score. There were no patients with an EHRA I score. One year after the procedure, 41 patients (71%) had an EHRA I score, 14 patients (24%) had an EHRA II score, and 3 patients (5%) had an EHRA III score. Four years after the procedure, 33 patients (57%) had an EHRA I score, 21 patients (36%) had an EHRA II score, and 4 patients (7%) had an EHRA III score. Ten years after surgery, 26 (45%) patients had an EHRA I score, 26 (45%) patients had an EHRA II score, and 6 (10%) patients had an EHRA III score (Figure 3). A statistically significant difference was observed in EHRA scores when comparing scores before and after one year and when comparing scores four and ten years post-surgery (*p* < 0.000). There was no statistically significant difference between the EHRA scores 1 year after and 4 years after the surgery (*p* = 0.126) and 4 and 10 years after surgery (*p* = 0.172).

## 4. Discussion

Despite scientific advances, treating atrial fibrillation remains a major challenge. Medication treatments are often ineffective for patients, and the side effects of medicines further reduce their quality of life [13,14]. There are several alternatives to medical treatment: per catheter treatments with cryothermia, radiofrequency energy, and pulsed-field therapy [14,15]. Surgical ablation also demonstrates higher freedom from AF than drug therapies [4].

Cox et al. first reported the Maze procedure in 1989 [16]. The classic Maze procedure is technique-dependent, with long operation times and high complication rates. Therefore, a series of modified Maze techniques have been developed. The Cox-Maze III procedure remains the gold standard treatment for patients with AF, though it remains technically demanding. Introducing new ablation technologies has made the procedure much easier and safer, making it more widely embraced [4]. The Wolf Mini Maze is the most famous minimally invasive modified Maze procedure [17]. Bipolar radiofrequency ablation is a safer and more efficient method for achieving transmural lesions than unipolar radiofrequency ablation [4].

Whether the procedure was effective was based on the absence of AF. A blanking period of 3 months after AF ablation procedures has been accepted in all studies because it can be difficult to identify true early recurrences from transient early arrhythmia recurrences related to electrical changes during the procedure [18]. We found that sinus rhythm is maintained in 70% of patients one year after the procedure and in 43% 10 years after the procedure. Most patients develop rhythm disturbances within a year after the procedure, with a slower increase in the number of rhythm disturbances after that. The rate of AF recurrence after the procedure varies depending on the centre where the surgery was performed. The overall rate of freedom from AF varies from 59% to 91%, averaging approximately 75% [19,20,21,22]. Several centres report the results for short-term follow-ups ranging from 6 to 12 months. Our ten-year period of patient tracking data is, therefore, unique. We also noticed an interesting trend: in the second year after surgery, there were fewer patients with rhythm disturbances. We used a three-month blanking period, but even after this, some patients continued to have transient rhythm disturbances for several months, which did not recur later [23]. These results were obtained for early AF recurrences occurring a few days to a week after the blanking period.

Ma N, Mei J, Lu RX, Jiang ZL, Tang M, and Ding FB report a high success rate for AF patients with previously failed catheter ablation history [24]. Therefore, one of the indications for selecting the Mini Maze was prior ineffective atrial fibrillation ablation. A total of 76% (44) of patients had a catheter ablation procedure before surgery. Mini Maze surgery helped to completely isolate the bilateral pulmonary vein and left atrial posterior wall with good quality and integrity of the ablation line. The left atrial appendage was also resected during surgery [24].

The Mini Maze procedure, although minimally invasive, is still a surgical procedure. The most commonly reported complications of the procedure are pulmonary vein stenosis, tamponade, pacemaker implantation, stroke, respiratory infections, renal failure, bleeding, and death. The literature describes mortality rates of less than 0.5% [25]. Surgical complications are relatively rare. A total of 1.7% of the procedures required a sternotomy to control bleeding. Other studies described surgical complications in 3.2% of cases, post-surgical complications in 3.2%, pulmonary complications in 2.1%, and cardiac complications in 2.6% [26]. A total of 1.4% of patients required permanent pacemaker implantation. There were no deaths in our centre after the Mini Maze procedure. However, two major complications were observed: a transient cerebral ischaemic attack and early post-operative bleeding, which led to a thoracotomy. Neurological symptoms were unexpressed and regressed within 24 h. As a result, the patient’s hospital stay was not prolonged. Two patients had a permanent pacemaker implanted in the early post-operative period. Other complications were related to respiratory infections treated with antibiotics.

Freedom from AF most often resulted in reduced symptoms, but symptom relief also occurred despite little effect on the arrhythmia [27]. Changes for the better in symptoms and quality of life have been observed even in patients with recurrent AF [27]. AF ablation may change the perception of AF so that patients may experience diminished symptoms despite recurring episodes of arrhythmia [13]. Significant impairment has been noted in well-being, physical and social functioning, and mental health [10]. Many patients with AF are of working age, so as their general well-being and mental and physical health improve, so does their working capacity.

Nevertheless, the benefits of surgery in patients with paroxysmal or persistent AF are unquestionable; there is still a lack of long-term follow-up data for such patients. Long-term follow-up multicentre studies are needed to assess the effectiveness of treatments and possible complications more accurately. This would help to improve the selection of patients for the procedures and their effectiveness. Studies show a good outcome in patients undergoing Mini Maze surgery after failed catheter ablation [24]. Therefore, this treatment could be used in clinical practice as a second-line treatment for such patients to prevent AF recurrence.

There were some limitations in this study. The first one is that this study is one single-centre experience. All data were derived retrospectively. We analysed a small number of patients. It was a non-randomised trial. Some patients could not be contacted for post-operative follow-up due to a change in their contact details, and some refused to come for follow-up visits due to the COVID-19 pandemic. Atrial fibrillation paroxysms were identified only via ECG and Holter monitoring, so some asymptomatic recurrence may have been missed. We were also unable to calculate the sex-specific variation in AF incidence and LA diameters because of the small number of women in this study.

## 5. Conclusions

Mini Maze procedures show good results in patients with ineffective previous catheter ablation or medical treatment. This surgical procedure has also been deemed safe. Despite the interventional nature of the procedure, complications have been observed with no lasting consequences. Although AF relapses increase with time, the impact on patients’ quality of life is long-term.

## Figures and Tables

**Table 1:** Preoperative characteristics of patients.

	Paroxysmal AF (*n* = 36)	Persistent AF (*n* = 22)	*p*-Value
Age	51.36 ± 9.19	49.73 ± 8.59	0.504
Female	5	4	0.98
CHA2DS2-VAsc Score 0	2	6	0.94
CHA2DS2-VASc Score 1	27	12	
CHA2DS2-VAsc Score 2	5	3	
CHA2DS2-VASc Score ≥ 3	2	1	
NYHA functional class I	13	8	0.946
NYHA functional class II	16	9	
NYHA functional class III	7	5	
LA area (cm^2^)	23.91 ± 4.11	26.77 ± 7.03	0.095
LA volume index (mL/m^2^)	38.89 ± 10.66	45.57 ± 14.50	0.05
LA inferior–superior diameter (mm)	59.2 ± 6.3	60.1 ± 8.9	0.671
LA medial–lateral diameter (mm)	46.6 ± 4.4	4.6 ± 5.4	0.636
AF duration (months)	68.84 ± 52	71.53 ± 49	0.414

**Table 2:** LA characteristics before surgery and 4 and 10 years after.

	Paroxysmal AF	Persistent AF	*p*-Value
LA area (cm^2^) before	23.91 ± 4.11	26.77 ± 7.03	0.095
LA area (cm^2^) 4 years after	27.72 ± 5.74	28.86 ± 6.08	0.76
LA area (cm^2^) 10 years after	30 ± 5.28	31.03 ± 6.17	0.50
LA inferior–superior diameter (mm) before	59.2 ± 6.3	60.1 ± 8.9	0.671
LA inferior–superior diameter (mm) 4 years after	64.5 ± 6.4	65.9 ± 7.5	0.463
LA inferior–superior diameter (mm) 10 years after	68.6 ± 5.8	69.5 ± 7.4	0.581
LA medial–lateral diameter (mm) before	46.6 ± 4.4	46.0 ± 5.4	0.636
LA medial–lateral diameter (mm) 4 years after	48.0 ± 5.3	50.2 ± 5.3	0.127
LA medial–lateral diameter (mm) 10 years after	50.0 ± 4.5	53.5 ± 5.8	0.016
LA volume index (mL/m^2^) before	38.89 ± 10.66	45.57 ± 14.50	0.05
LA volume index (mL/m^2^) 4 years after	46.22 ± 14.64	50.20 ± 10.54	0.29
LA volume index (mL/m^2^) 10 years after	51.02 ± 12.04	59.58 ± 13.45	0.016

**Table 3:** LA characteristics before surgery and 4 and 10 years after, according to recurrence of AF.

	No Recurrence of AF	Recurrence of AF	*p*-Value
LA area (cm^2^) before	23.72 ± 5.2	25.84 ± 4.59	0.192
LA area (cm^2^) 4 years after	26.83 ± 5.33	29.96 ± 6.91	0.02
LA area (cm^2^) 10 years after	29.34 ± 3.4	31.79 ± 6.07	0.073
LA inferior–superior diameter (mm) before	58.6 ± 7.5	60.7 ± 7.0	0.249
LA inferior–superior diameter (mm) 4 years after	63.0 ± 6.1	67.3 ± 7.0	0.027
LA inferior–superior diameter (mm) 10 years after	67.0 ± 6.1	71.2 ± 6.1	0.020
LA medial–lateral diameter (mm) before	45.6 ± 4.2	47.2 ± 4.9	0.209
LA medial–lateral diameter (mm) 4 years after	47.7 ± 5.7	50.0 ± 4.9	0.116
LA medial–lateral diameter (mm) 10 years after	52.9 ± 5.1	59.9 ± 4.9	0.027
LA volume index (mL/m^2^) before	27.63 ± 10.54	30.50 ± 15.41	0.544
LA volume index (mL/m^2^) 4 years after	42.13 ± 9.94	56.89 ± 16.06	0.000
LA volume index (mL/m^2^) 10 years after	51.06 ± 12.22	58.21 ± 13.44	0.035

## Data Availability

The data presented in this study are available on request from the corresponding author. The data are not publicly available due to ethical requirements.
